# Dibenzazecine compounds with a novel dopamine/5HT_2A _receptor profile and 3D-QSAR analysis

**DOI:** 10.1186/1471-2210-6-11

**Published:** 2006-09-15

**Authors:** Alexandra Hamacher, Mathias Weigt, Michael Wiese, Barbara Hoefgen, Jochen Lehmann, Matthias U Kassack

**Affiliations:** 1 Department of Pharmaceutical Chemistry, Institute of Pharmacy, University of Bonn, An der Immenburg 4, 53121 Bonn, Germany; 2 Institute of Pharmacy, University of Jena, Philosophenweg 14, 07743 Jena, Germany

## Abstract

**Background:**

Antipsychotics are divided into typical and atypical compounds based on clinical efficacy and side effects. The purpose of this study was to characterize *in vitro *a series of novel azecine-type compounds at human dopamine D_1_-D_5 _and 5HT_2A _receptors and to assign them to different classes according to their dopamine/5HT_2A _receptor profile.

**Results:**

Regardless of using **affinity **data (p*K*_i _values at D_1_-D_5 _and 5HT_2A_) or **selectivity **data (15 log (K_i _ratios)), principal component analysis with azecine-type compounds, haloperidol, and clozapine revealed three groups of dopamine/5HT_2A _ligands: 1) haloperidol; 2) clozapine plus four azecine-type compounds; 3) two hydroxylated dibenzazecines. Reducing the number of K_i _ratios used for principal component analysis from 15 to two (the D_1_/D_2 _and D_2_/5HT_2A _K_i _ratios) obtained the same three groups of compounds. The most potent dibenzazecine clustering in the same group as clozapine was the non-hydroxylated LE410 which shows a slightly different D_2_-like receptor profile (D_2L _> D_3 _> D_4.4_) than clozapine (D_4.4 _> D_2L _> D_3_). The monohydroxylated dibenzacezine LE404 clusters in a separate group from clozapine/LE410 and from haloperidol and shows increased D_1 _selectivity.

**Conclusion:**

In conclusion, two compounds with a novel dopamine/5HT_2A _receptor profile, LE404 and LE410, with some differences in their respective D_1_/D_2 _receptor affinities including a validated pharmacophore-based 3D-QSAR model for D_1 _antagonists are presented.

## Background

Dopamine is an important neurotransmitter in the mammalian CNS which has influence on physiological, behavioural and neuroendocrine functions, mediated through receptors on the cell surface. Five different dopamine receptor subtypes have been cloned and characterized. They belong to the super-family of G protein-coupled receptors (GPCR) and can be divided into two subfamilies, D_1_-like (D_1_, D_5_) and D_2_-like (D_2_, D_3_, D_4_) receptors, according to their sequence homologies, biochemical properties, and pharmacologic profiles [1]. D_1_-like receptor stimulation activates adenylyl cyclase (AC) via coupling to stimulatory G protein Gα_s_/Gα_olf _subunits leading to an increase in intracellular cAMP concentrations. In contrast, D_2_-like receptors are Gα_i_/Gα_o _linked and inhibit AC activity [2]. Dopamine receptors are clinically important drug targets for the treatment of disorders such as Parkinson's disease and schizophrenia [3]. Blockade of dopamine D_2 _receptors is the main feature of antipsychotic action. Typical antipsychotics like the first generation D_2 _receptor antagonists haloperidol or chlorpromazine can cause therapy-limiting extrapyramidal-motor side effects (EPS). Second generation (atypical) antipsychotics are serotonin/dopamine antagonists with no or low EPS at doses showing antipsychotic activity and have significantly greater affinity for 5HT_2A _than for D_2 _receptors [4]. This serotonin-dopamine ratio could contribute to atypicality [5-7] but further investigations are needed to define the precise mechanism of atypical antipsychotics. However, antipsychotic activity is not only the result of D_2 _and 5HT_2A _receptor blockade but an inhibitory/modulating effect on various dopamine and serotonin (D_1_, D_2_, D_3_, D_4_, 5HT_1A_, 5HT_1D_, 5HT_2A_, 5HT_2C_) and further receptors [8]. Within the heterogeneous group of atypical antipsychotics, only clozapine exhibits effects against treatment-resistant schizophrenia [9]. Responsible for this net effect among atypical antipsychotics may be the moderate affinity of clozapine at various receptor subtypes, especially at D_1_-receptors. A dysfunction in D_1_-receptor modulation in the prefrontal cortex contributes to the negative symptoms and cognitive deficits observed in schizophrenia. However, selective D_1 _antagonism alone has not turned out as an effective antipsychotic principle [9,10].

**Figure 1:**
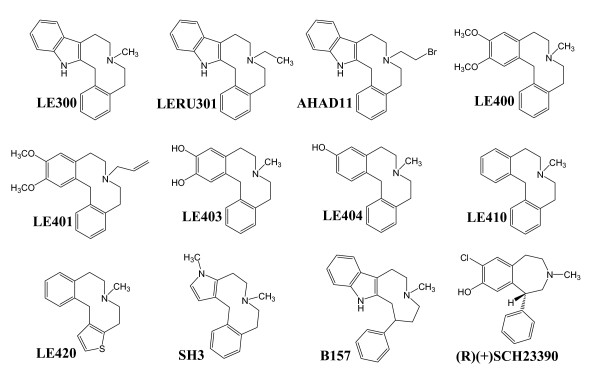
Structural formulas of the indolobenzazecine LE300, SCH23390, and a series of ten derived compounds.

LE300, an indolobenzacezine (figure 1) has previously been characterized [11] and shows a binding profile similar to that of clozapine, however with a greater affinity for D_1_- than D_2_-like receptors. A series of LE300-derived compounds was recently synthesized and screened at dopamine D_1_, D_2L_, and D_5 _receptors by a previously published functional calcium assay [12,13]. The aim of the current study was to investigate the comprehensive binding and functional receptor profile of the most active of the dibenzazecine derivatives of LE300 (LE400, LE401, LE403, LE404, LE410, and LE420, figure 1) at all human dopamine and 5HT_2A _receptors, to test whether data from the calcium assay initially used for screening of LE300-derived compounds [13] correlate with other assays measuring functional activation of GPCRs (cAMP, [^35^S]-GTPγS), and to establish a 3D-QSAR pharmacophore model of these ligands. Heterologous competition binding experiments were carried out at recombinantly expressed human dopamine and 5HT_2A _receptors, and obtained data were compared with functional data from intracellular [cAMP] and [Ca^2+^] measurements and [^35^S]-GTPγS-binding. Indeed, dibenzazecine compounds with a previously not available receptor profile (increased antagonist activity at D_1_-like and 5HT_2A _receptors) were found. 3D-QSAR studies were performed resulting in QSAR models allowing further rational ligand design at a molecular level.

## Results

### Receptor expression and characterization

**Figure 2:**
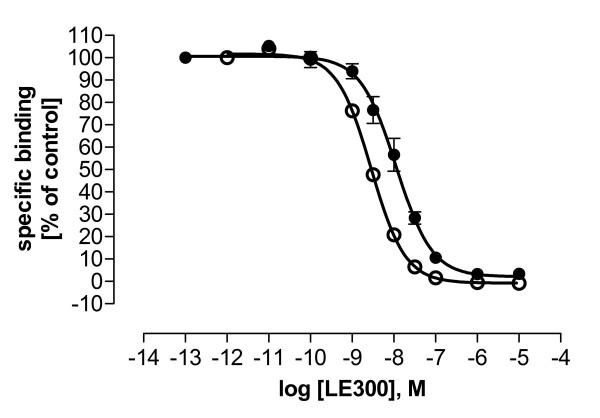
**Buffer-dependent differences in hD_1 _receptor potencies of LE300 in competition binding**. Inhibition by LE300 of the binding of 0.2 nM [^3^H]SCH23390 to hD_1 _receptor expressing HEK293 cell membranes using Krebs-HEPES buffer pH 7.4 (●) or TRIS-HCl pH 7.4 (○), respectively. Hill slopes were not different from unity. Nonspecific binding was determined with 1 μM SCH23390, and was less than 7%. Data shown are mean ± SEM, n = 3. K_i _(Krebs-HEPES): 10.0 ± 1.15 nM; K_i _(TRIS-HCl): 2.36 ± 0.13 nM.

Homologous radioligand competition binding experiments were performed to determine the receptor expression levels (B_max_) and binding affinities (K_d_) of the used radioligands. Average B_max _and K_d _values for each receptor are shown in table 1. All K_d _values except for 5HT_2A _receptors were 3-6-fold higher than those found in the literature (table 1, [14-17]). This effect could be attributed to the use of isotonic Krebs-HEPES-buffer pH 7.4 in this study instead of the widely used TRIS-HCl buffer pH 7.4 in the literature. Figure 2 shows as an example the buffer-dependent inhibition by LE300 of [^3^H]SCH23390 binding to D_1 _receptor membranes. Using Krebs-HEPES instead of TRIS-HCl buffer yielded ~4-fold higher K_i _values of LE300 (figure 2) but allowed a better comparison of functional and binding data. A buffer-dependent change of affinity was also observed with the test compounds. However, the K_d _ratios among the receptor subtypes using Krebs-HEPES buffer were equal to literature data using TRIS-HCl (not shown).

**Table 1:** Characterization of recombinantly expressed human dopamine and h5HT_2A _receptors in HEK293 cell membrane preparations

	K_d_	B_max_	K_d *Literature*_^a)^
	
Receptor	nM	fmol/mg protein	nM
hD_1_	1.93 ± 0.24	3520 ± 790	0.35
hD_2L_	0.18 ± 0.02	1641 ± 462	0.06
hD_3_	0.84 ± 0.10	4060 ± 973	0.275
hD_4.4_	0.30 ± 0.06	493 ± 83.7	0.09
hD_5_	1.50 ± 0.23	1030 ± 263	0.30
h5HT_2A_	0.54 ± 0.07	165 ± 84.2	0.91

^a) ^Data taken from [14-17]

[^3^H]SCH23390 was used for D_1_-like, [^3^H]spiperone for D_2_-like and 5HT_2A _receptors in homologous competition experiments. Data are mean ± SEM, n ≥ 3.

### Radioligand binding studies

**Figure 3:**
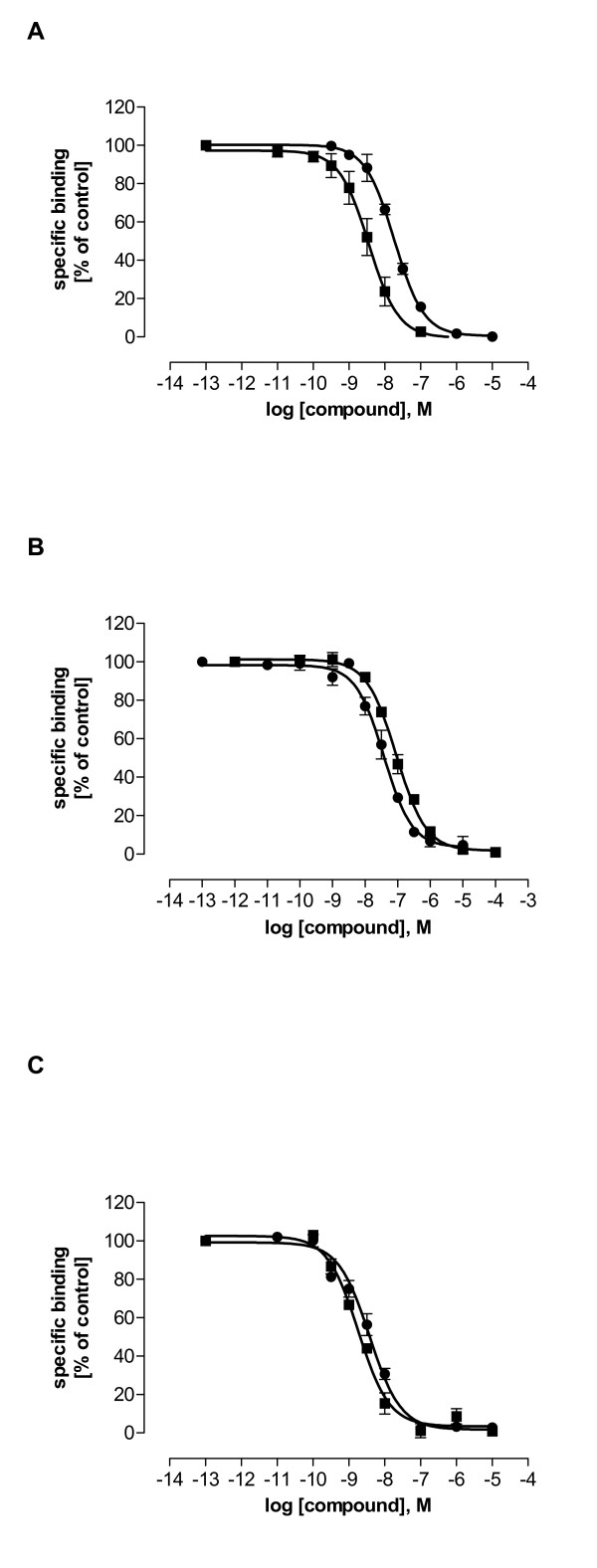
**Heterologous competition binding curves of LE404 (■) and LE410 (●) at hD_1 _(A), hD_2L _(B), and 5HT_2A _(C) receptors**. Data shown are the means ± SEM of specific binding of at least four determinations assayed in triplicate. **A**. 0.2 nM [^3^H]SCH23390 was used for hD_1 _receptors. Nonspecific binding was determined with 1 μM LE300. **B**. 0.1 nM [^3^H]spiperone was used for hD_2L _receptors. Nonspecific binding was determined with 1 μM haloperidol. **C**. 0.1 nM [^3^H]spiperone was used for h5HT_2A _receptors. Nonspecific binding was determined with 1 μM ketanserin.

Binding affinities of the compounds LE300, LE400, LE401, LE403, LE404, LE410, and LE420 (figure 1) were estimated at recombinant dopamine and 5HT_2A _receptors in cell membrane preparations. Further compounds used for 3D-QSAR analysis of D_1 _receptor ligands (AHAD11, B157, LERU301, SH3, figure 1) were tested at D_1 _receptors only due to limited availability. For the sake of comparison, haloperidol as a classical antipsychotic, clozapine as an atypical antipsychotic, and LE300 were included as reference compounds. Figure 3 shows the radioligand displacement curves of the most potent hD_1 _and hD_2L _ligands LE404 and LE410 at hD_1 _(**A**), hD_2L _(**B**), and 5HT_2A _(**C**) receptors. p*K*_*i *_values are displayed in table 2 (LE300, LE400, LE401, LE403, LE404, LE410, and LE420) and table 3 (AHAD11, B157, LERU301, SH3).

**Table 2:** Characterization of compounds by heterologous competition binding

Compound	p*K*_i_					
	
	hD_1_	hD_2L_	hD_3_	hD_4.4_	hD_5_	h5HT_2A_
Haloperidol	6.55 ± 0.09	8.56 ± 0.05	8.00 ± 0.05	8.10 ± 0.04	7.50 ± 0.06	6.84 ± 0.12
Clozapine	6.68 ± 0.03	6.60 ± 0.06	6.13 ± 0.05	6.93 ± 0.08	6.50 ± 0.08	8.23 ± 0.07
LE300	7.98 ± 0.06	7.19 ± 0.04	6.48 ± 0.04	6.46 ± 0.08	7.99 ± 0.05	9.65 ± 0.04
LE400	5.58 ± 0.16	5.90 ± 0.05	5.28 ± 0.07	4.79 ± 0.06	5.44 ± 0.07	6.86 ± 0.13
LE401	4.77 ± 0.25	5.06 ± 0.13	4.83 ± 0.16	< 4^a)^	4.79 ± 0.50	< 4^a)^
LE403	7.94 ± 0.06	6.43 ± 0.07	6.14 ± 0.10	6.26 ± 0.06	7.84 ± 0.05	8.40 ± 0.08
LE404	8.47 ± 0.10	7.10 ± 0.05	6.73 ± 0.06	7.23 ± 0.03	8.53 ± 0.09	8.79 ± 0.07
LE410	7.76 ± 0.04	7.54 ± 0.06	6.86 ± 0.07	6.32 ± 0.06	7.78 ± 0.10	8.40 ± 0.10
LE420	6.89 ± 0.07	6.64 ± 0.05	6.07 ± 0.06	5.83 ± 0.11	6.92 ± 0.04	7.97 ± 0.05

^a) ^Displacement of radioligand was < 30% at 10 μM

Haloperidol, clozapine, and LE compounds were characterized at dopamine and h5HT_2A _receptors. [^3^H]SCH23390 was used for hD_1_-like and [^3^H]spiperone for hD_2_-like and h5HT_2A _receptors. Displayed are p*K*_i _values ± SEM, n ≥ 3.

**Table 3:** Characterization of AHAD11, B157, LERU301, and SH3 at hD_1 _receptors used for 3D-QSAR analysis.

	Compound			
	AHAD11	B157	LERU301	SH3
	
p*K*_*i *_(hD_1_)	5.82 ± 0.07	6.98 ± 0.05	7.26 ± 0.03	6.17 ± 0.04

Displayed are p*K*_*i *_values ± SEM, n ≥ 3.

All compounds showed similar affinities at hD_1 _and hD_5 _receptors. The mono-hydroxylated LE404 turned out as the most potent compound at hD_1_/hD_5 _receptors with p*K*_*i *_values of 8.47 and 8.53, respectively, followed by the bis-hydroxylated LE403 which is 3-10-fold less potent than LE404. Replacement of the hydroxy- by methoxy-substituents resulting in LE400 dramatically decreased the affinity at all tested receptors. An increase of the size of the nitrogen substituent (allyl group of LE401) further decreased the affinity at all tested receptors. Except LE400 and LE401, all other compounds possessed up to 33fold (LE403, LE404) higher affinities for D_1_-like than for D_2_-like receptors (table 2). Among D_2_-like receptors, all compounds – except LE404 – showed the highest affinity at hD_2L _and lower affinities at hD_3 _and hD_4.4 _receptors similar to the profile of haloperidol at D_2_-like receptors. However, different to haloperidol which shows a strong D_2 _over D_1 _selectivity, LE compounds (except LE400 and LE401) show selectivity for D_1 _over D_2_. Removal of the hydroxy-group of LE404 yielding LE410 resulted in a dramatic loss of D_1 _over D_2 _selectivity, and left LE410 as the most potent compound at hD_2L _and hD_3 _receptors with p*K*_*i *_values of 7.54 and 6.86, respectively. Bioisosteric replacement of one benzene residue in LE410 by thiophene gave LE420 showing a similar receptor profile as LE410 but with reduced affinity at all tested receptors. LE404 displayed a receptor profile within the D_2_-like receptors which is unique among the tested LE compounds. Within the D_2_-like receptors, LE404 reached the highest affinity at hD_4.4 _(p*K*_*i*_: 7.23), a slightly lower affinity at hD_2L _(p*K*_*i*_: 7.10), and the lowest affinity at hD_3 _receptors (p*K*_*i*_: 6.73). The D_2_-like receptor affinity pattern of LE404 is thus similar to clozapine (D_4.4 _≥ D_2L _> D_3_). In contrast to clozapine which appeared ~ equipotent at D_1_/D_2 _receptors in all of our test systems, LE404 shows 25fold selectivity for D_1 _over D_2_. LE404 displayed higher affinities than LE300 at all dopamine receptors except hD_2L _where both compounds are ~ equipotent. All compounds except LE401 showed the highest affinities among all tested receptors at 5HT_2A_. The most potent compound at 5HT_2A _was LE300 with an affinity in the subnanomolar range followed by LE404 in the low nanomolar range. LE300, LE400, LE403, LE404, LE410, and LE420 achieved K_i-D2_/K_i-5HT2A _selectivity ratios > 7.

### Functional studies (cAMP, Ca^2+ ^and [^35^S]-GTPγS binding) at hD_1 _and hD_2L _receptors

**Figure 4:**
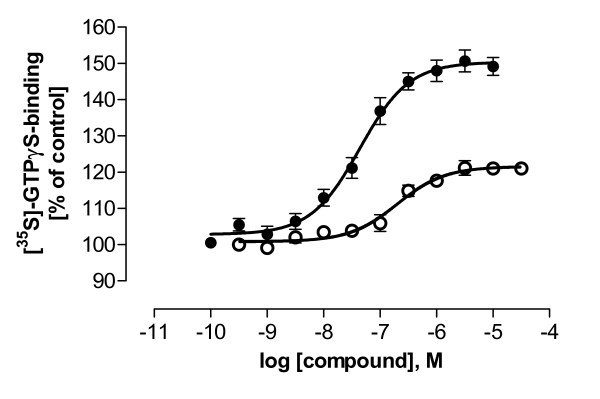
**hD_1 _receptor stimulation of [^35^S]-GTPγS-binding by dihydrexidine (●) and SKF38393 (○)**. Both agonists were used in HEK293 cell membranes recombinantly expressing hD_1 _receptors in the presence of 1 μM GDP. Data shown are means ± SEM of at least three experiments.

For functional studies, hD_1 _and hD_2L _receptors were chosen as characteristic representatives of each of the two dopamine receptor subtype groups allowing a comparison of functional and binding data. The inhibition by LE compounds of agonist-induced changes in intracellular [cAMP] and [Ca^2+^] in intact HEK293 cells, and [^35^S]-GTPγS binding in HEK293 cell membranes were estimated. Table 4 shows EC_50 _and IC_50 _values of standard ligands at D_1 _and D_2L _receptors. The effects of LE compounds on [^35^S]-GTPγS binding were determined in the presence of agonist. At hD_1 _receptors the full agonist dihydrexidine [18] was used for [^35^S]-GTPγS binding experiments instead of SKF38393 which was used in cAMP and Ca^2+ ^studies. In membrane preparations from HEK293-hD_1 _cells, dihydrexidine showed a significantly higher increase in [^35^S]-GTPγS binding than SKF38393 (figure 4). Dihydrexidine gave an EC_50 _of 43.8 ± 8.23 nM (hD_1_, figure 4). A difference between dihydrexidine and SKF38393 was not observed in intracellular [Ca^2+^] and [cAMP] measurements (data not shown), and thus SKF38393 was used in Ca^2+ ^and cAMP studies. The EC_50 _of quinpirole at hD_2L _receptors was estimated as 437 ± 93.1 nM (data not shown). All LE compounds except LE401 showed an inhibition of [^35^S]-GTPγS binding between 25 and 40% (not shown).

**Table 4:** EC_50 _and IC_50 _values of reference compounds at D_1 _and D_2L _receptors in functional studies.

		Agonist		Antagonist	
			
Assay	Receptor	SKF38393	Quinpirole	LE300	Haloperidol
[cAMP]	hD_1_	33.0 ± 4.01		123 ± 31.1	
	hD_2L_		9.61 ± 3.31		1.54 ± 0.39
[Ca^2+^]	hD_1_	24.5 ± 4.19		718 ± 168	
	hD_2L_		8.62 ± 2.66		0.30 ± 0.10

Data shown are EC_50_/IC_50 _values in nM ± SEM, n ≥ 3.

**Figure 5:**
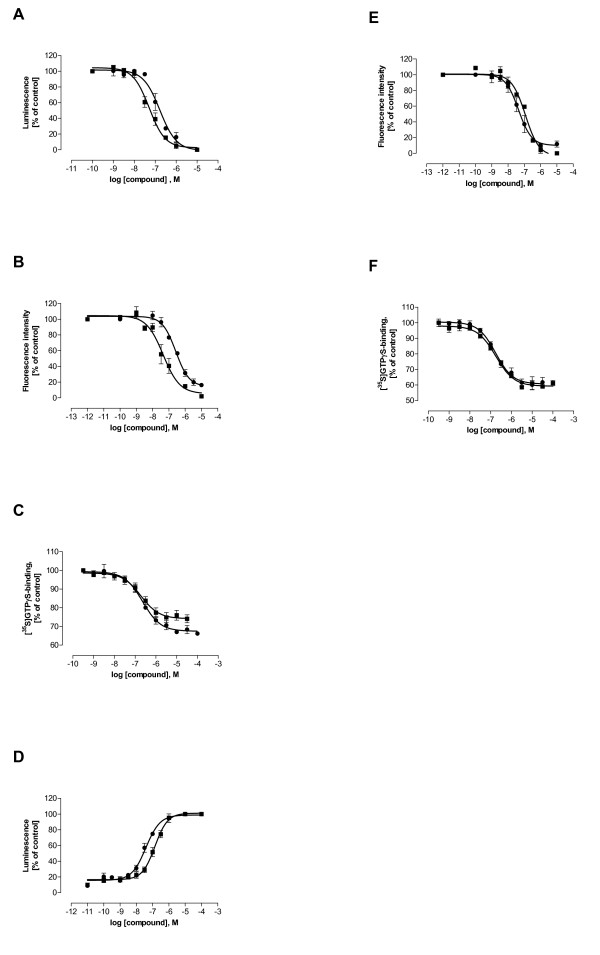
**Functional characterisation of LE404 (■) and LE410 (●) at hD_1 _(A, B, C) and hD_2L _receptors (D, E, F)**. **A **Inhibition by LE404 and LE410 of 100 nM SKF38393-stimulated accumulation of intracellular [cAMP]. Data shown are means ± SEM of at least four determinations assayed in triplicate. **B **Inhibition by LE404 and LE410 of 100 nM SKF38393-stimulated increase in intracellular [Ca^2+^]. Data shown are means ± SEM of at least four determinations assayed in triplicate. **C **Inhibition by LE404 and LE410 of G-protein activation obtained by 1 μM dihydrexidine-stimulation. Data shown are means ± SEM of two independent experiments assayed in duplicate. **D **Inhibition by LE404 and LE410 of 100 nM quinpirole-stimulated decrease of intracellular [cAMP] in the presence of 10 μM forskolin. Data shown are means ± SEM of at least four determinations assayed in triplicate. **E **Inhibition by LE404 and LE410 of 30 nM quinpirole-stimulated increase in intracellular [Ca^2+^]. Data shown are means ± SEM of at least four determinations assayed in triplicate. **F **Inhibition by LE404 and LE410 of G-protein activation obtained by 10 μM quinpirole-stimulation. Data shown are means of two independent experiments assayed in duplicate.

None of the tested compounds (neither LE compounds nor reference compounds haloperidol or clozapine) showed any agonist effect in functional studies (data not shown). All test compounds inhibited agonist-stimulated effects on intracellular [cAMP] and [Ca^2+^] and on [^35^S]-GTPγS binding at D_1 _or D_2L _receptors, respectively, in a concentration-dependent manner. LE400 in Ca^2+ ^studies and LE401 in all functional assays achieved ≤ 50% inhibitory activity at 10 μM. Concentration-inhibition curves of the most potent novel compounds at D_1 _and D_2L _receptors, LE404 and LE410, are displayed in figure 5. Apparent functional p*K*_*i *_values (p*K*_*i app*_) derived from inhibition experiments of all compounds in cAMP, Ca^2+^, and [^35^S]-GTPγS studies are presented in table 5. When comparing p*K*_*i *_values of one compound from cAMP, Ca^2+^, and [^35^S]-GTPγS studies, differences may occur (e.g., clozapine at D_1 _receptors: p*K*_*i*_(cAMP): 6.46; p*K*_*i *_([^35^S]-GTPγS): 7.47) but also good accordance was observed (e.g., LE404 at D_1 _receptors: p*K*_*i *_values between 7.95 and 8.20). The rank orders of potency of the tested compounds at D_1 _and D_2L _receptors, respectively, remained similar for the three functional assays: the most potent compound at D_1 _receptors in all three functional assays (table 5) and in binding (table 2) is LE404 whereas the weakest compounds are LE400 and LE401. At D_2L _receptors, LE300, LE410, and LE404 are the most potent compounds after haloperidol whereas again, LE400 and LE401 are the weakest (binding: table 2, functional assays: table 5). LE404 has a 25-fold selectivity for D_1 _over D_2L _receptors based on binding (table 2). This D_1 _preference was lost in cAMP and [^35^S]-GTPγS experiments (LE404 is ~ equipotent at D_1 _and D_2L_) but a certain D_1 _preference (3-fold) was retained in Ca^2+ ^studies (table 5). Haloperidol showing a 100-fold D_2L _over D_1 _selectivity in binding (table 2) retained this 100-fold D_2L _selectivity in [^35^S]-GTPγS experiments but showed an increased D_2L _selectivity in cAMP and Ca^2+ ^studies (> 1000-fold). LE410 which displayed an only moderate D_1 _selectivity in binding (~2-fold, table 2) became D_2L _selective in cAMP and Ca^2+ ^studies but was ~ equipotent in [^35^S]-GTPγS binding. These results show that cAMP and Ca^2+ ^studies uprate the potency of compounds at D_2L _compared to D_1 _receptors (tables 2 and 5).

**Table 5:** Inhibitory potencies of the LE compounds on agonist-induced effects on [cAMP]_i_, [Ca^2+^]_i_, and [^35^S]-GTPγS binding

	p*K*_*i app*_					
	
Compound	[cAMP]_i_		[Ca^2+^]_i_		[^35^S]-GTPγ S binding	
			
	hD_1_	hD_2L_	hD_1_	hD_2L_	hD_1_	hD_2L_
Haloperidol	6.80 ± 0.10	9.88 ± 0.07	6.61 ± 0.09	10.0 ± 0.13	7.10 ± 0.91	9.10 ± 0.07
Clozapine	6.46 ± 0.05	7.30 ± 0.07	6.54 ± 0.15	6.92 ± 0.11	7.47 ± 0.29	7.48 ± 0.08
LE300	7.55 ± 0.13	8.73 ± 0.10	7.22 *± *0.15	7.93 ± 0.12	7.75 ± 0.12	8.14 ± 0.11
LE400	5.35 ± 0.17	6.88 ± 0.09	< 5.00^a)^	< 5.00^a)^	6.25 ± 0.13	6.39 ± 0.14
LE401	5.00 ± 0.13	< 5.00^a)^	< 5.00^a)^	< 5.00^a)^	< 5.00^a)^	< 5.00^a)^
LE403	7.02 ± 0.09	7.23 ± 0.12	7.57 ± 0.11	7.14 ± 0.08	7.48 ± 0.12	7.20 ± 0.15
LE404	7.95 ± 0.09	8.01 ± 0.08	8.20 ± 0.15	7.71 ± 0.01	8.10 ± 0.13	8.13 ± 0.08
LE410	7.35 ± 0.12	8.63 ± 0.07	7.39 ± 0.07	8.13 ± 0.11	8.02 ± 0.08	8.13 ± 0.09
LE420	6.44 ± 0.21	7.69 ± 0.08	6.73 ± 0.09	7.08 ± 0.12	7.17 ± 0.11	7.51 ± 0.08

^a) ^Inhibitory activity was ≤ 50% at 10 μM.

Concentration-effect curves were obtained with hD_1 _and hD_2L _receptors. Data shown are apparent p*K*_*i *_values (p*K*_*i app*_) ± SEM, n ≥ 3.

### Statistical comparison of functional and binding data at D_1 _and D_2L _receptors

The multiple intercorrelation and thus the equality of the results obtained by binding and the three functional assays at D_1 _and D_2L _receptors, respectively, was determined by principal component analysis (PCA). Results of the PCA comparing the four test systems (factor loadings) are displayed in table 6. The first extracted principal component (PC) for D_1 _receptors described 89.8% of the total variance among the four p*K*_*i *_variables (cAMP, Ca^2+^, [^35^S]-GTPγS, and binding) with factor loadings > 0.91 (table 6) leaving an eigenvalue of only 0.237 for the second PC. For D_2L _receptors, the first extracted PC explained 97.5% of the total variance among the four p*K*_*i *_variables (factor loadings > 0.98, table 6) leaving an eigenvalue of only 0.050 for the second PC. Following the idea that a PC with an eigenvalue of << 1 has no legitimacy for the description of the total variance [19], the PCA results indicate a significant multiple correlation among the four variables for D_1 _and D_2L _receptors, respectively.

**Table 6:** Factor loadings of the four variables used in principal component analysis

**Variable**	**hD**_1_	**hD**_2L_
**cAMP**	0.953	0.985
**Ca**^2+^	0.955	0.986
**[**^35^**S]-GTPγS**	0.913	0.985
**radioligand binding**	0.970	0.994

### Nature of antagonism of LE compounds at D_1 _and D_2 _receptors

**Figure 6:**
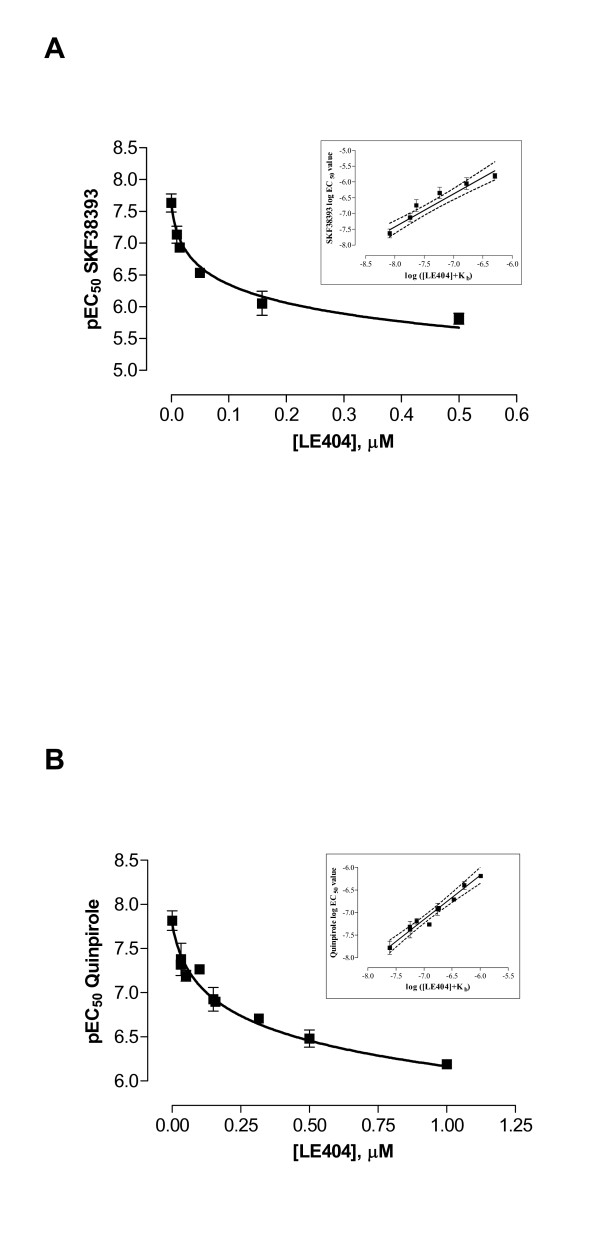
**Functional analysis of the antagonist effect of LE404 at hD_1 _and hD_2L _receptors**. The analysis was carried out by measuring the attenuation by LE404 of the agonist-induced increase in intracellular [Ca^2+^] in HEK293 cells recombinantly expressing hD_1 _and hD_2L _receptors, respectively. Dashed lines show 95% confidence intervals. SKF38393 was used as agonist at hD_1 _receptors, quinpirole at hD_2L_. All slopes were not significantly different from unity. Presented data are means ± SEM from at least three independent experiments each with at least threereplicates. **A, B**. Clark analysis of LE404 at hD_1 _(**A**) and hD_2L _receptors (**B**). Inserts show Clark plots.

Next, the nature of antagonism of LE compounds at D_1 _and D_2L _receptors was tested by Clark analysis [20]. Since LE404 was the most potent compound at D_1 _and LE410 the most potent at D_2L _receptors (binding, table 2), LE404 and LE410 were chosen as representatives to undergo functional analysis for competitive antagonism. In the presence of increasing concentrations of LE404 and LE410, parallel rightward shifts of the agonist concentration-effect curves in the Ca^2+ ^assay were observed without loss of maximum efficacy at hD_1 _and hD_2L _receptors (data not shown). The rightward shift of the concentration-effect curves of the agonist was analyzed with non-linear regression analysis according to Lew and Angus [20]. Data were fitted to equations (1) and (2) (see methods). An F-Test showed no significant difference (p > 0.2), thus equation (2) with a Hill slope of 1 was the preferred model and used to obtain p*K*_*b *_values. Results for LE404 at hD_1 _and hD_2L _receptors are presented in figures 6A and 6B. Inserts show the Clark plots (mean log EC_50 _values of the agonist concentration-effect curves plotted against log ([LE404] + K_b_) which yielded straight lines at both receptor subtypes. p*K*_*b *_values were calculated as: hD_1_: p*K*_*b *LE404 _= 8.09 ± 0.15; p*K*_*b *LE410 _= 7.69 ± 0.13; hD_2L_: p*K*_*b *LE404 _= 7.61 ± 0.10; p*K*_*b *LE410 _= 8.05 ± 0.11. p*K*_*b *_values of LE404 and LE410 derived from non-linear Clark analysis show no significant difference to those derived from Schild analysis [21] (data not shown). Both functional analyses (Schild, Clark) give thus evidence for a competitive antagonistic behaviour of LE404 and LE410 at D_1 _and D_2L _receptors.

### Statistical analysis of binding affinities and selectivities at dopamine and 5HT_2A _receptors

In order to perform a statistically valid test for the discovery of ligands with differing affinity profiles at dopamine D_1_-D_5 _and 5HT_2A _receptors among the examined compounds, multiple intercorrelations of binding **affinity **values (p*K*_*i*_, table 2) as well as binding **selectivity **values [log (K_i _ratio) = log (K_i Receptor 1_/K_i Receptor 2_)] were investigated in two separate PCA's. PCA has already successfully been applied to define similar and deviating responses among biological data (variables) [22,23]. LE401 was excluded from both PCA's because precise p*K*_*i *_values were missing at hD_4.4 _and h5HT_2A _receptors (table 2). In the first PCA, eight compounds were examined (haloperidol, clozapine, LE300, LE400, LE403, LE404, LE410, and LE420) for their affinity in six test systems (D_1_-D_5 _and 5HT_2A _receptors). The PCA resulted in two PC's from which the first extracted 80.5% of the total variance among the eight p*K*_*i *_variables, and the second extracted 11.4%. The factor loadings of the eight variables (compounds) are listed in table 7 and show that the eight compounds define three subgroups of dopamine/5HT_2A _ligands: 1) clozapine, LE300, LE400, LE410, and LE420 with factor loadings contributing to the first PC of > 0.739; 2) haloperidol in the second PC with a factor loading of -0.923; 3) LE403 and LE404 in the second PC with opposite direction to haloperidol (factor loadings 0.868 and 0.886) indicating that LE403 and LE404 display an affinity profile opposite to that of haloperidol. For the second PCA, for each of the eight compounds, log (K_i _ratio) values [= log (K_i Receptor 1_/K_i Receptor 2_)] were calculated for all possible 15 receptor affinity ratios (D_1_/D_2L_, D_1_/D_3_, D_1_/D_4.4_, D_1_/D_5_, D_1_/5HT_2A_, D_2L_/D_3_, D_2L_/D_4.4_, D_2L_/D_5_, D_2L_/5HT_2A_, D_3_/D_4.4_, D_3_/D_5_, D_3_/5HT_2A_, D_4.4_/D_5_, D_4.4_/5HT_2A_, D_5_/5HT_2A_) using the data from table 2. The resulting log (K_i _ratio) data matrix contains **selectivity **information for each of the compounds. Results of this second ("selectivity") PCA were basically identical to results from the first ("affinity") PCA (table 7). The first extracted PC explained 74.8% of the total variance among the eight variables (log (K_i _ratio) values), and the second PC extracted 15.4% of the total variance. The second ("selectivity") PCA discovered the same three subgroups of dopamine/5HT_2A _ligands as did the first PCA: 1) clozapine, LE300, LE400, LE410, and LE420 with factor loadings contributing to the first PC of > 0.780 (table 7); 2) haloperidol in the second PC with a factor loading of -0.901; 3) LE403 and LE404 in the second PC with opposite direction to haloperidol (factor loadings 0.933 and 0.893). Thus, regardless of using **affinity **information (p*K*_*i*_) or **selectivity **information (log (K_i _ratio)) for PCA, the same three subgroups of dopamine/5HT_2A _ligands were discriminated. The agreeing results from both PCA's underline that the statistical analysis of binding affinities and selectivities at dopamine and 5HT_2A _receptors did not create chance correlations.

**Table 7:** PCA results of affinity and selectivity data at dopamine and 5HT_2A _receptors

Variable	p*K*_*i*_		log (K_i Receptor 1_/K_i Receptor 2_)	
		
	1^st ^Principal Component	2^nd ^Principal Component	1^st ^Principal Component	2^nd ^Principal Component
Haloperidol	-0.205	**-0.923**	-0.272	**-0.901**
Clozapine	**0.796**	0.260	**0.780**	0.356
LE300	**0.775**	0.629	**0.921**	0.354
LE400	**0.955**	0.222	**0.983**	0.122
LE403	0.488	**0.868**	0.290	**0.933**
LE404	0.410	**0.886**	0.395	**0.893**
LE410	**0.739**	0.568	**0.840**	0.433
LE420	**0.829**	0.549	**0.873**	0.474

The first PCA is using p*K*_*i *_values from table 2 (**affinity **information), the second log (K_i _ratio) values (**selectivity **information). Log (K_i _ratio) values [= log (K_i Receptor 1_/K_i Receptor 2_)] were calculated for all possible 15 receptor affinity ratios using data from table 2. Displayed are factor loadings for the first two PC's after Varimax rotation.

### 3D-QSAR (CoMFA/CoMSIA studies)

**Figure 7:**
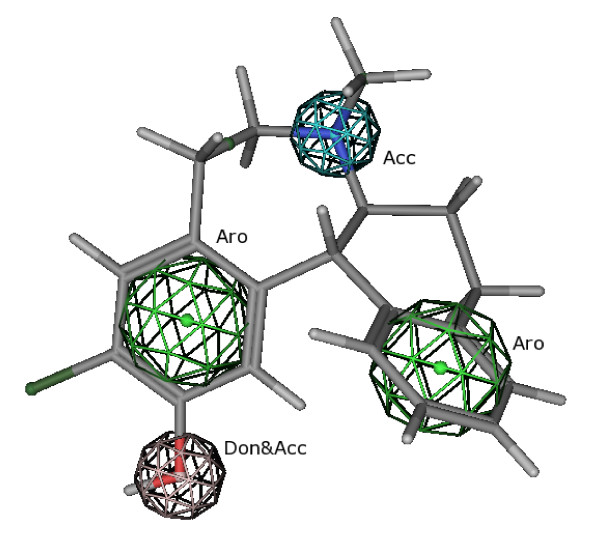
**3D model of (-)-2b-SCH39166 with H-donor/acceptor and aromatic features**. This model was used as pharmacophore-template for the LE compounds.

**Figure 8:**
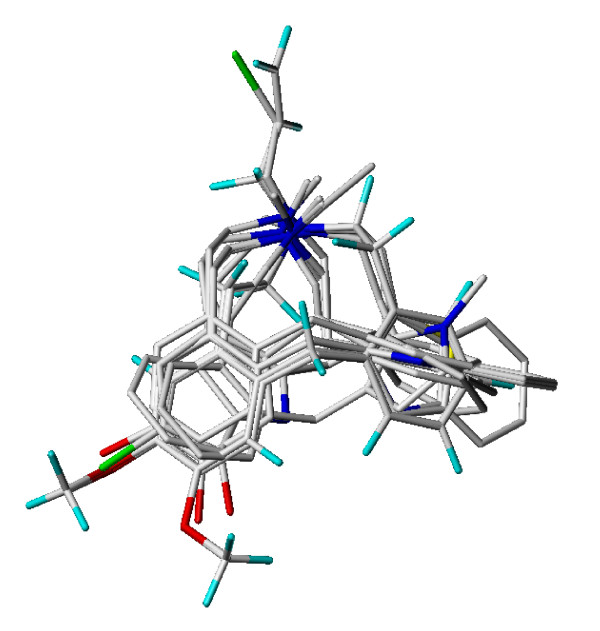
Alignment of the final 3D-QSAR models of the LE compounds.

**Figure 9:**
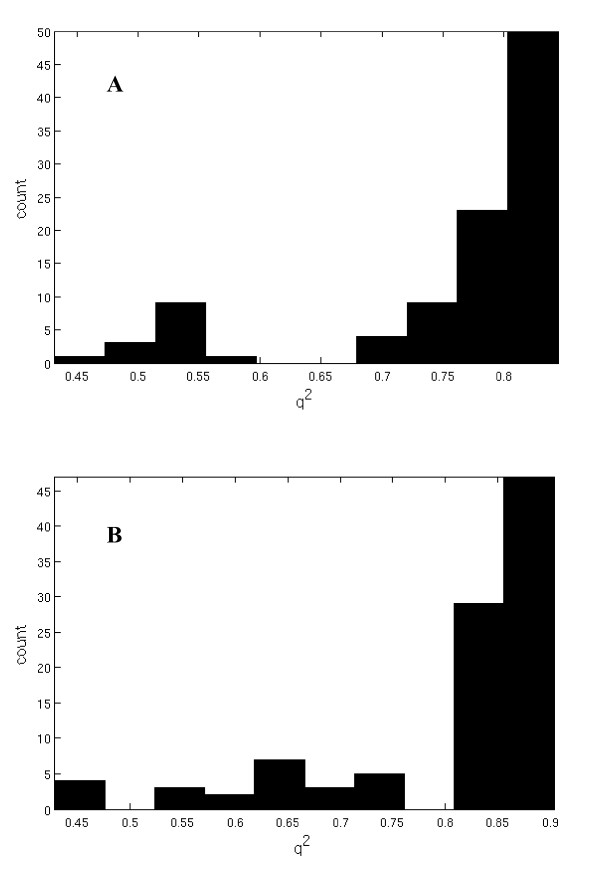
**Validation of the final alignment models using the random groups PLS method ("leave-many-out")**. **A**. CoMFA field. **B**. CoMSIA field.

Since the main feature of the LE compounds is their D_1 _selectivity, a 3D-QSAR pharmacophore model for the D_1 _receptor was establish using the 12 compounds shown in figure 1 and their D_1_-p*K*_*i *_values from table 2 and 3. For a successful CoMFA/CoMSIA study, it is crucial to find an appropriate alignment of the examined compounds. It is not necessary that all compounds possess the bioactive conformation but it is useful that the compounds adopt a relative conformation and position to each other as they would bind to the receptor. The D_1_/D_5 _selective antagonist (-)-2b-SCH39166 (ecopipam) was taken as a pharmacophore template. (-)-2b-SCH39166 is a benzonaphthazepine, a rigid analogue of SCH23390, thus limiting the number of possible conformations (figure 7) [24]. Unfortunately, (-)-2b-SCH39166 was not available to us for testing, and was thus not used for the final QSAR-analysis. However, due to its rigid nature, it was helpful to find a good starting point for selecting conformations and alignments of the 12 compounds from figure 1. Essential pharmacophore features of (-)-2b-SCH39166 are the two aromatic rings and the basic nitrogen (hydrogen acceptor) while the hydroxyl group served as an optional H-donor/acceptor feature (figure 7). Results of the alignment of the final models of the LE compounds are shown in figure 8. The aromatic residues and basic nitrogen atoms remain the main pharmacophore features. Crossvalidation results (leave-one-out) for the final models for CoMFA and CoMSIA both using steric and electrostatic fields are displayed in table 8, and show crossvalidation parameters q^2 ^of 0.82 for CoMFA and 0.88 for CoMSIA. To prove that these models were not a result of a chance correlation, a stability test was performed using the random groups PLS method ("leave-many-out"). The test showed a high stability of the models presented in figure 8 with a mean q^2 ^of 0.76 (SD 0.10) for the combined steric and electrostatic field in CoMFA and a mean q^2 ^of 0.81 (SD 0.12) in CoMSIA. The distribution of the q^2 ^values for this validation is shown in figure 9.

**Table 8:** Crossvalidation results for the final alignment models of the LE compounds

**Field**	**Minimum σ**	**No of components**	SDEP*	**q**^2^
CoMFA	0.75	3	0.60	0.82
CoMSIA	0.75	3	0.50	0.88

* SDEP: standard error of prediction

The models are displayed in figure 8 using steric and electrostatic fields for both CoMFA and CoMSIA.

## Discussion

Among a group of new azecine compounds, this study has revealed two dibenzacezines (LE404 and LE410) with potent activity at dopamine and 5HT_2A _receptors displaying a novel receptor profile at D_1_-D_5 _and 5HT_2A _receptors. Compounds were evaluated in binding studies at D_1_-D_5 _and 5HT_2A _receptors and functionally (cAMP, Ca^2+^, [^35^S]-GTPγS) at D_1 _and D_2L _receptors, representative for the two subgroups of G_s _(D_1_-like) and G_i _(D_2_-like) coupled dopamine receptors. PCA revealed the equivalence of functional and binding p*K*_*i *_values (table 6) even though binding, cAMP, Ca^2+^, and [^35^S]-GTPγS assays differ strongly in the applied conditions (equilibrium: binding, cAMP, [^35^S]-GTPγS; non-equilibrium: Ca^2+^) and used endpoints (competition binding, G protein activation, second [cAMP] and "third" [Ca^2+^] messenger generation). A comparison of p*K*_*i *_values of one compound in the four different assays thus leads to differences, e.g., K_i _ratios of haloperidol at D_1_/D_2L _receptors are ~1200 in cAMP, ~2500 in Ca^2+^, and ~100 in [^35^S]-GTPγS and binding studies but the rank order of potency remains almost unchanged (tables 2 and 5). Mottola et al. [25] have introduced the term "functional selectivity" to propose that depending on the experimental (buffer, equilibrium) and cellular conditions regarding receptor and G protein expression, a mixture of agonist/partial agonist and/or antagonist actions are likely. The ~2-fold difference in D_1 _and D_2L _receptor expression in this study (table 1) may thus contribute to differences in p*K*_*i *_values observed in functional and binding studies. The same reasons may serve as an explanation for differences in the K_d _values of SCH23390 and spiperone in this study and in the literature (table 1) and for the ~1.4–5.5-fold differences in the affinity of LE300 in this and a previous study [11]. Further, affinities in this study were tested at recombinantly expressed receptors in HEK293 cell membranes in Krebs-HEPES-buffer whereas the previous study used CHO cell membranes in a Tris-Mg^2+^-buffer [11]. As was shown in figure 2, different buffers can result in significantly different affinity of a ligand.

LE404 and LE410 are competitive antagonists as was shown by Clark analysis (figure 6). p*K*_*b *_values of LE404 and LE410 derived from these functional analyses are in accordance with p*K*_*i *_values derived from inhibition curves (tables 5 and 2). Statistical analysis (PCA) of binding **affinity **data (p*K*_*i *_values, table 2) and binding **selectivity **data [log (K_i _ratio) values, calculated from table 2] resulted in three groups of ligands: first: haloperidol; second: clozapine, LE300, LE400, LE410, and LE420; and – interestingly – a third group: containing LE403 and LE404 (table 7). The most potent compounds in group 2 and group 3 are LE410 and LE404. LE410 has a similar affinity profile as clozapine except the lower potency of LE410 at the hD_4.4 _receptor (table 2). In contrast, LE404 has a 25-fold selectivity for D_1 _over D_2L _receptors and thus a novel dopamine/5HT_2A _receptor profile. Interestingly, if instead of **all **K_i _ratio values which have been used for the PCA in table 7 only the D_1_/D_2L _and D_2L_/5HT_2A _ratios of all compounds were used for clustering, the same three groups were found: 1) haloperidol, 2) clozapine, LE400, LE410, LE420, LE300, and 3) LE403 and LE404 (table 9). Thus, instead of six receptors and 15 K_i _ratios, a reduction to three receptors (D_1_, D_2L_, 5HT_2A_) and two K_i _ratios is sufficient to obtain the same clustering of compounds.

**Table 9:** K_i _ratio values (K_i-D1_/K_i-D2 _and K_i-D2_/K_i-5HT2A_) of all test compounds except LE401

**Compound**	**K**_**i-D1**_**/K**_**i-D2**_	**K**_**i-D2**_**/K**_**i-5HT2A**_
**Haloperidol**	102 (100^a)^)	0.02 (0.05^a)^)

**Clozapine**	0.83 (1.58^a)^)	42.6 (19.95^a)^)
**LE400**	2.09	9.12
**LE410**	0.60	7.24
**LE420**	0.56	21.4
**LE300**	0.16	294

**LE403**	0.03	93.5
**LE404**	0.04	49.0
**RMI-81582**	0.05^a)^	31.6^a)^

**SCH23390**	0.0004^b)^	50.0^c)^

^a) ^Value is calculated from [6].

^b) ^Value is calculated from [33,34].

^c) ^Value is calculated from [34,35].

Further, data for SCH23390 and RMI-81582 are calculated from literature data. K_i _values used are derived from radioligand binding studies.

Meltzer et al. suggested the use of D_1_/D_2L _and D_2L_/5HT_2A _ratios to allow a clustering of antipsychotics into typical and atypical compounds [5-7]. However, instead of Meltzer et al. who calculated p*K*_*i *_ratio values which are imprecise in defining selectivity (same selectivity may result in different p*K*_*i *_ratios depending on the potency), K_i _ratios (table 9) or log (K_i _ratio) values (for PCA in table 7) were calculated in this study. K_i _ratios recalculated from data of Meltzer et al. [5] and K_i _ratios from this study were no more different than 3-fold (table 9). LE300, LE403, LE404, LE410, and LE420 achieved K_i-D2_/K_i-5HT2A _selectivity ratios > 7 which may suggest an atypical behaviour of these compounds according to Meltzer et al. [5]. However, so far there are no *in vivo *behavioural studies underlying an antipsychotic effect of the LE compounds. The third group of ligands, LE403 and LE404, differ from LE410 by a 15-20-fold increase in D_1 _selectivity (table 9). RMI-81582 has very similar D_1_/D_2 _and D_2_/5HT_2A _K_i _ratios as LE403 and LE404 (table 9) and was shown to exert antipsychotic effects [26]. A further increase in D_1 _selectivity over D_2_, e.g., compound SCH23390 (table 9), results in a complete loss of antipsychotic activity [5,9,10]. Therefore, LE403 and LE404 might display an antipsychotic effect which however needs to be proven in *in vivo *studies. Only *in vivo *studies take into account the complexity of neuropsychiatric diseases including expression, distribution, and regulation of multiple receptors as well as adaptive processes.

This study confirmed recent findings that an increase in the size of the residue of the azecine nitrogen is detrimental to the affinity at dopamine/5HT_2A _receptors (table 2) [11]. Hydroxylated versus non-hydroxylated dibenzacezines differ in their affinity and selectivity profiles (LE410, LE404, table 2) and define 2 separate groups. Monohydroxylation (LE404) results in higher potency than bis-hydroxylated compounds (LE403). Abolishing the H-donor properties by exchanging hydroxyl by methoxy groups was detrimental to the potency (LE400 versus LE403). Binding data of all compounds in figure 1 have been used to establish a valid 3D-QSAR pharmacophore model for D_1 _receptors (figure 8). The resulting model shows excellent q^2 ^values for crossvalidation results and random groups PLS tests for both, CoMFA and CoMSIA (figure 9) excluding a chance correlation. The pharmacophore model is thus a solid basis for further improvement of dopamine receptor ligands.

## Conclusion

In conclusion, this study has revealed two compounds, the dibenzacezines LE410 and LE404 with a novel dopamine/5HT_2A _receptor profile. LE404 and LE410 differ in their D_1_/D_2L _selectivity. LE410 clusters in one group with the atypical antipsychotic clozapine but has a different D_2_-like receptor profile (hD_2L _> hD_3 _> hD_4.4_) than clozapine (hD_4.4 _> hD_2L _> hD_3_). LE404 clusters in a separate group from clozapine/LE410 and from haloperidol and shows increased D_1 _selectivity similar to the experimental compound RMI-81582 which displayed antipsychotic activity [26]. An antipsychotic activity of LE404 and LE410 in *in vivo *studies still needs to be shown. Further, a validated 3D-QSAR pharmacophore model for D_1 _antagonists is presented.

## Methods

### Materials

LE300, 400, 401, 403, 404, 410, and 420 were synthesized according to methods previously published [11,13]. [^3^H]SCH23390 (66.0 Ci/mmol), [^3^H]spiperone (118 Ci/mmol), and [^35^S]-GTPγS were obtained from Amersham Biosciences (Buckinghamshire, UK). SKF38393 was purchased from TOCRIS (Bristol, U.K.). A pRc/CMV vector construct for hD_3 _receptors was kindly provided by Dr. P. Sokoloff (Paris, France) [27] and a pcDNA3.1+ construct containing cDNA coding for the h5HT_2A _receptor was obtained from the UMR cDNA resource center [28]. All other reagents were supplied by Sigma Chemicals unless otherwise stated.

### Cell culture

HEK293 cells stably expressing hD_1_, hD_2L_, or hD_5 _dopamine receptors were established as previously described [11,29]. Stable cell lines of HEK293 cells (ATCC, Rockville, MD, USA) were generated by transfecting the plasmids coding for hD_3 _and h5HT_2A _using polyfect^® ^transfection reagent (Qiagen, Hilden, Germany) according to the manufacturer's instructions and were selected using G-418 (400 μg/ml medium). All stably transfected cell lines were grown in Dulbecco's modified Eagle Medium Nutrient Mixture F-12 Ham (DMEM/F12 1:1 mixture) containing 10% fetal bovine serum, 100 μg/ml streptomycine, 100 U/ml penicillin G, 5 mM L-glutamine, and 200 μg/ml active G-418. The human D_4.4 _receptor was stably expressed in CHO cells (kindly provided by Dr. van Tol, Toronto, Canada) and grown in Ham F12 medium supplemented with 10% fetal bovine serum, 100 μg/ml streptomycin, 100 U/ml penicillin G, 1 mM L-glutamine, and 200 μg/ml active G-418. Cells were incubated at 37°C in a humidified atmosphere under 5% CO_2_.

### Membrane preparation

Confluent 145 mm tissue culture dishes (Greiner Bio-One, Frickenhausen, Germany) of HEK293 or CHO cells were harvested by scraping, resuspended in ice-cold Krebs-HEPES buffer (118 mM NaCl, 4.7 mM KCl, 1.2 mM MgSO_4_, 1.2 mM KH_2_PO_4_, 4.2 mM NaHCO_3_, 11.7 mM D-Glucose, 1.3 mM CaCl_2_, 10 mM HEPES, pH 7.4), and disrupted using a Polytron homogenizer on ice (Kinematica AG, Basel, Switzerland). After centrifugation at 40,000 × g at 2°C, the supernatant was discarded, and pellet was washed twice with ice-cold Krebs-HEPES buffer. Eventually, the pellet was resuspended in the appropriate binding buffer (see below) and stored in aliquots at -80°C until use for radioligand binding. The method of Bradford [30] was used to determine the protein content of membrane preparations with bovine serum albumin as standard.

For [^35^S]-GTPγS-binding, cell pellets were resuspended in 10 mM Tris-HCl/1 mM EDTA, pH 7.4, homogenized in a glass-teflon homogenizer and centrifuged for 15 min (40,000 × g, 4°C). Supernatant was discarded, and pellet was washed twice with ice-cold Tris-HCl/EDTA buffer and finally resuspended in 50 mM Tris-HCl, 4 mM MgCl_2_, 1 mM EDTA, 250 mM sucrose, pH 7.4, and stored at -80°C. The protein content was determined according to the Bradford method [30] with gamma immunoglobulin as standard.

### Radioligand binding experiments

The equilibrium dissociation constants K_d _of the radioligands used ([^3^H]SCH23390 for hD_1_-like, [^3^H]spiperone for hD_2L_-like and h5HT_2A _receptors) were determined in homologous competition binding experiments and receptor densities of the respective dopamine receptor cell membrane preparations (B_max _values) were calculated using the DeBlasi equation [31]. Heterologous competition binding experiments were performed in Krebs-HEPES buffer in a final volume of 1.1 ml at 26°C for 2 h (D_1_-like receptors) or 3 h (D_2_-like receptors and 5HT_2A _receptors) as described previously [11]. Cell membranes (total protein amount ~90 μg/tube) were incubated with 0.2 nM final [^3^H]SCH23390 (D_1_-like receptors) or with 0.1 nM final [^3^H]spiperone (D_2_-like receptors and 5HT_2A _receptors) and competing drugs. The assay was terminated by rapid filtration of 1 ml through polyethylene imine pretreated (0.2%) glass fiber filters (Schleicher und Schuell, Dassel, Germany), followed by two washes with ice-cold distilled water. Filters were soaked in 5 ml of scintillation fluid for at least 12 h and bound radioactivity was determined by liquid scintillation counting. Nonspecific binding of [^3^H]SCH23390 was determined in the presence of 1 μM LE300, nonspecific binding of [^3^H]spiperone in the presence of 1 μM haloperidol for hD_2L_-like receptors and 1 μM ketanserin for h5HT_2A _receptors.

### Estimation of [^35^S]-GTPγS-binding in HEK293 membranes

Cell membranes (for hD_1_: total protein amount ~16 μg, ~1.5 pmol receptor/mg protein; for hD_2L_: total protein amount ~16 μg, ~0.3 pmol receptor/mg protein) were incubated with test compounds, 1 μM GDP, agonist (1 μM dihydrexidine for hD_1_, 10 μM quinpirole for hD_2L_) and 100 pM [^35^S]-GTPγS in microplates in a total volume of 200 μl assay buffer (20 mM HEPES-NaOH (pH 7.4), 100 mM NaCl, 3 mM MgCl_2_). Plates were incubated for 60 min at 30°C. Reaction was terminated by rapid vacuum filtration through GF/C filter plates (PerkinElmer), and filter plates were washed four times with 200 μl Tris-HCl (pH 7.4). Radioactivity retained on the filter plates was counted in a microplate counter (Microbeta, PerkinElmer).

### Measurement of changes in intracellular [Ca^2+^] in HEK293 cells

Measurement of changes in intracellular [Ca^2+^] was performed as previously described using a NOVOstar microplate reader with a built-in pipetor (BMG LabTech, Offenburg, Germany) [11]. HEK293 cells expressing the respective dopamine receptor were loaded with 3 μM Oregon Green 488 BAPTA-1/AM (Molecular Probes, Eugene, OR) for 1 h at 25°C in Krebs-HEPES buffer containing 1% Pluronic F-127. Then, cells were rinsed three times with Krebs-HEPES buffer containing 0.5% bovine serum albumin, diluted, and evenly plated into 96 well plates (Greiner, Frickenhausen, Germany) at a density of ~35,000 cells/well. Microplates were kept at 37°C. Fluorescence intensity was measured at 520 nm (bandwidth 35 nm) for 5 s at 0.4 s intervals to monitore baseline. Buffer alone or test compounds dissolved in buffer were then injected into separate wells, and fluorescence intensity was monitored at 520 nm (bandwidth 35 nm) for 25 s at 0.4 s intervals. Excitation wavelength was 485 nm (bandwidth 12 nm). Concentration-inhibition curves in the presence of the test compounds were obtained by pre-incubating the cells with the compounds for 30 min at 37°C prior to injection of agonist (hD_1_: 100 nM SKF38393; hD_2L_: 30 nM quinpirole).

### Measurement of changes in intracellular [cAMP] in HEK293 cells

Intracellular [cAMP] levels were estimated by using a cAMP reporter gene assay. pCRE-Luc Cis-Reporter plasmid (Path Detect^® ^CRE Cis-Reporting System, Stratagene, La Jolla, CA) coding for the firefly luciferase under the control of a cAMP response element was transiently transfected in HEK293 cells stably expressing the hD_1 _or hD_2L _receptor. 24 h after transfection, cells were reseeded in poly-L-lysine-coated (Biochrom, Berlin, Germany) white 96-well plates with clear bottom (Greiner, Frickenhausen, Germany) at a density of ~25,000 cells/well. Microplates were incubated for 48 h at 37°C and 5% CO_2 _before using the cells for adenylyl cyclase stimulation or inhibition experiments. Cells were then exposed to increasing concentrations of test compounds dissolved in serum-free and phenol red-free medium and incubated for 3 h at 37°C and 5% CO_2_. In case of hD_2L_, 10 μM forskolin was added. Antagonistic activity was tested by pre-incubation of test compounds for 30 min at 37°C and 5% CO_2 _prior to the addition of agonist (hD_1_: 100 nM SKF38393; hD_2L_: 100 nM quinpirole plus 10 μM forskolin) for 3 h. Incubation was terminated by adding 100 μl of cell lysis buffer (8 mM tricine, 1 mM dithiothreitol, 2 mM EDTA, 5 % Triton^® ^X-100, pH 7.8) for 20 min at 4°C. Luciferase activities were measured with the LUMIstar microplate reader (BMG LabTech, Offenburg, Germany). After monitoring the baseline for 0.3 s, 100 μl of luciferase assay reagent (30 mM tricine, 0.5 mM ATP, 10 mM MgSO_4_, 0.5 mM EDTA, 10 mM dithiothreitol, 0.5 mM coenzyme A, 0.5 mM D-luciferin, pH 7.8) was added and luminescence was measured at 25°C for 12.7 s at 0.1 s intervals. Luminescence was corrected by subtracting baseline levels.

### Functional analysis of antagonism

Functional analysis of the antagonist effect of LE404 and LE410 was carried out by measuring the attenuation by LE404 or LE410 of the agonist-induced increase in intracellular [Ca^2+^] in HEK293 cells recombinantly expressing hD_1 _or hD_2L _receptors. At least four antagonist concentrations were used. Functional data were used for nonlinear regression analysis according to Clark [20]. The pEC_50 _values of the agonist curves were plotted against the concentration of test compounds LE404 or LE410 and analyzed by non-linear regression curve fitting using the following equations:

(1) pEC_50 _= -log ([B]^n ^+ 10^-pKb^) - log c

(2) pEC_50 _= -log ([B] + 10^-pKb^) - log c

where [B] is the concentration of antagonist (LE404 or LE410), p*K*_*b *_is the negative decadic logarithm of the antagonist dissociation constant, n the Hill coefficient, and log c the difference between the p*K*_*b *_and the pEC_50 _value of the agonist concentration-response curve in absence of the antagonist. Fits to equations (1) and (2) were compared by an F-test.

### Data analysis

Radioligand-binding and functional data (measurement of intracellular [Ca^2+^], [cAMP], and [^35^S]GTPγS binding) were analyzed by fitting the pooled data from at least three experiments (each with three replicates) to the four parameter logistic equation using Prism software 3.0 from GraphPad (GraphPad Software; San Diego, CA, USA). Competition-binding experiments were fitted best to a one-site binding model. Inhibition constants K_i _from radioligand binding competition experiments were calculated from IC_50 _values using the Cheng-Prusoff equation [32]. Apparent functional K_i _values were calculated according to the following equation adapted from Cheng and Prusoff [32]:

K_i _= IC_50_/(1+L/EC_50_)

where IC_50 _is the inhibitory concentration of the antagonist to block by 50% the agonist effect, EC_50 _is the effective concentration 50% of the used agonist (i.e., SKF38393 for hD_1_, and quinpirole for hD_2L _receptors), and L is the molar concentration of the used agonist. Data (data points in figures and numbers in tables) are given as mean ± SEM of at least three independent experiments each performed with triplicates unless otherwise stated. Statistical analyses including principal component analysis were performed using SPSS (version 12.0.1 for Windows).

### 3D-QSAR (CoMFA/CoMSIA) studies

All calculations were carried out on an x86-compatible PC running SuSE-Linux 9.2. For molecular modelling, SYBYL 7.0 (Tripos Inc., St. Louis, Missouri, USA) and MOE 2004.03 for Linux (Chemical Computing Group Inc., Montreal, Quebec, Canada) were used. Conformational clustering was done using MATLAB Release 13 for Linux (The MathWorks Inc., Natick, MA, USA). Conformational analyses of all 12 compounds from figure 1 were done using a repeated molecular dynamics based simulated annealing approach as implemented in SYBYL 7.0. MMFF94 served as the force field with distance dependent electrostatics. A molecule was heated up to 1000 K within 2000 fs, held at this temperature for 2000 fs and annealed to 0 K for 10000 fs using an exponential annealing function. By applying this procedure, a total of 100 conformations were sampled during 100 cycles to account for conformational flexibility and to find the most likely conformations occurring most often in the resulting pool. This was done for both configurations of the protonated nitrogen atom because molecular mechanics is not able to switch configurations. All conformations were then optimized with the semi-empirical quantum mechanics method AM1 as implemented in MOPAC 6 from SYBYL and further compared using the SYBYL MATCH algorithm. Subsequently, a MATLAB clustering algorithm was used to extract the most divergent conformations using the root mean square (RMS) values of the comparison and the AM1 heat of formation. The most diverse and most often represented conformations of each compound were selected and overlaid with the pharmacophore resulting from the rigid ligand (-)-2b-SCH-39166 using the program MOE. The best 2–4 matched alignments per compound were selected for the CoMFA/CoMSIA study upon minimum RMS criteria and visual examination. These conformations were transferred to a SYBYL database and used as an initial alignment for the CoMFA/CoMSIA study. During an automated procedure, all possible combinations were tested on the CoMFA and CoMSIA combined steric/electrostatic fields with partial least squares analysis (PLS). In subsequent PLS analyses, the alignment was refined and the CoMFA/CoMSIA models were optimized. To prove that these models were not a result of a chance correlation, a stability test was performed using the random groups PLS method. Within this method, cross-validation was done with groups of more than one compound, which were excluded earlier during the model-building regression. Unlike the leave-one-out cross-validation, these groups are selected on a random basis and instead of twelve cross-validation groups, only five were used. Because of the random selection of the group members, this cross-validation was repeated a hundred times.

## Abbreviations

CHO, Chinese hamster ovary; CoMFA, comparative molecular field analysis; CoMSIA, comparative molecular similarity indices analysis; HEK, human embryonic kidney; PC, principal component; PCA, principal component analysis; RMI 81582, 2-chloro-11-(3-dimethylaminopropylidene)morphanthridine; SCH23390, *R*-(+)-7-chloro-8-hydroxy-3-methyl-1-phenyl-2,3,4,5-tetrahydro-1*H*-3-benzazepine; (-)-2b-SCH39166, (-)-trans-6,7,7a,8,9,13b-hexahydro-3-chloro-2-hydroxy-N-methyl-5H-benzo [d]naptho-(2,1-b)-azepine; SKF38393; (±)-1-phenyl-2,3,4,5-tetrahydro-(1*H*)-3-benzazepine-7,8-diol.

## Authors' contributions

AH established recombinant cell lines, carried out functional measurements and radioligand binding studies, and performed data evaluation. Further, AH drafted the manuscript. MWeigt carried out the 3D-QSAR studies. MWiese provided intellectual input and critical interpretation of the data. BH carried out calcium measurements. JL provided the LE compounds. MUK carried out principal component analyses and finalized the manuscript for publication. All authors read and approved the final manuscript.
